# Efficacy of three-dimensional electroanatomic mapping-guided minimally invasive transaxillary atrial appendage resection for focal atrial tachycardia originating from the atrial appendage in children

**DOI:** 10.3389/fped.2026.1852142

**Published:** 2026-06-24

**Authors:** Lulu Wang, Weitao Zhang, Liyun Zhao, Feng Ai, Xiaosong Hu, Jiangzhen Li, Taibing Fan, Shubo Song

**Affiliations:** Pediatric Heart Center, Fuwai Central China Cardiovascular Hospital (Huazhong Fuwai Hospital Affiliated to Zhengzhou University), Zhengzhou, China

**Keywords:** atrial appendage resection, atrial appendage tachycardia, atrial tachycardia, children, pulsed field ablation, radiofrequency catheter ablation, tachycardia-induced cardiomyopathy

## Abstract

**Objectives:**

To investigate the clinical characteristics and short-to-medium-term outcomes of minimally invasive transaxillary atrial appendage resection guided by the Carto3 three-dimensional electroanatomic mapping system for focal atrial tachycardia originating from the atrial appendage (atrial appendage tachycardia, AAT) in children.

**Methods:**

This single-center retrospective study consecutively enrolled 27 children with atrial tachycardia (AT) who underwent Carto3-guided electrophysiologic study between January 2023 and April 2026; 6 (22.2%) were confirmed to have AAT. Treatment was individualized according to anatomical origin: minimally invasive transaxillary appendage resection for appendage tip origin or aneurysm (*n* = 5) and pulsed field ablation (PFA) for appendage base origin (*n* = 1). The results for the two groups are reported separately.

**Results:**

All six patients (four male, 66.7%; mean age 10.28 ± 3.49 years) showed Carto3 activation patterns suggestive of focal origin. The earliest activation was at the appendage tip in 5 (83.3%) and at the base in 1 (16.7%); 5 (83.3%) had incessant AT; and 3 (50%) had tachycardia-induced cardiomyopathy (TIC). Appendage resection group (*n* = 5): All patients achieved immediate AT termination and sinus rhythm restoration. At a median follow-up of 13 months (IQR 6.75–16.25; minimum 6 months), no recurrence or procedure-related complications were observed. Given the very small sample size and limited follow-up, these preliminary findings should not be considered confirmatory evidence of safety and efficacy. PFA group (*n* = 1): Immediate success was achieved, but AT recurred at 1 month post-procedure (Holter-confirmed); the patient is currently being managed medically. In the three patients with TIC, LVEF improved from 46.3 ± 8.3% to 62.0 ± 6.1% and FS from 23.3 ± 4.8% to 33.3 ± 4.6% at the final follow-up, showing a descriptive improving trend; LVEDd decreased from 47.3 ± 3.5 mm to 42.0 ± 5.3 mm. Given *n* = 3, these results are presented descriptively, without inferential interpretation.

**Conclusions:**

In this small preliminary series, one-stop minimally invasive transaxillary appendage resection showed initial feasibility and favorable short-term outcomes in pediatric AAT with Carto3-confirmed appendage tip origin or associated aneurysm refractory to medical therapy. TIC secondary to AAT may be reversible after arrhythmia elimination. These preliminary findings require validation in larger, multicenter, prospective studies. The role of PFA in pediatric AAT warrants further investigation.

## 1 Introduction

Pediatric focal atrial tachycardia (AT) accounts for 10%–15% of supraventricular arrhythmias in children (1). Among its subtypes, atrial appendage tachycardia (AAT) is the most prevalent, with an incidence of 42%–50% (2). AAT may occur at any age, including during the fetal period, and typically presents with incessant tachycardia and non-specific symptoms. Persistent, uncontrolled AAT results in tachycardia-induced cardiomyopathy (TIC) in approximately 44.3% of cases (3).

Conventional antiarrhythmic drug therapy is largely ineffective in AAT. Although radiofrequency catheter ablation (RFCA) is recommended as the first-line treatment, the complex anatomy of the atrial appendage, particularly in the setting of appendage aneurysm, renders endocardial ablation technically challenging, with published acute failure rates of 44%–45.7% and post-ablation recurrence rates of 25.5% (3).

Minimally invasive surgical approaches, including transaxillary mini-thoracotomy and video-assisted thoracoscopic surgery (VATS), have demonstrated potential advantages over sternotomy in terms of reduced trauma, improved cosmesis, and accelerated recovery. Pulsed field ablation (PFA), a non-thermal energy modality based on irreversible electroporation, demonstrates tissue selectivity (4) that may enhance procedural safety in anatomically sensitive regions such as the atrial appendage.

This study analyzed the baseline characteristics, procedural findings, and follow-up outcomes of six pediatric patients with Carto3-confirmed AAT, aiming to provide a preliminary evaluation of the feasibility and short-to-medium-term clinical outcomes of minimally invasive transaxillary atrial appendage resection in children with AAT and to inform individualized treatment strategies.

## 2 Methods

### 2.1 Study population

This single-center retrospective study involves screening all consecutive children with AT who underwent Carto3 three-dimensional electroanatomic mapping (Biosense Webster, Johnson & Johnson, Diamond Bar, CA, USA) at the Pediatric Heart Center of Fuwai Central China Cardiovascular Hospital between January 2023 and April 2026. Of the 27 patients screened, 6 (22.2%) were confirmed to have AAT and met all the inclusion criteria; all were consecutively enrolled without exclusion. The data cutoff was 31 January 2026, and the last follow-up was 31 March 2026; no patients were lost to follow-up. This study was approved by the Institutional Review Board, and written informed consent was obtained from the guardians of each patient.

The inclusion criteria were as follows: (1) age <18 years; (2) AT confirmed by intracardiac electrophysiologic study and Carto3 activation mapping to originate from the atrial appendage (LAA or RAA); and (3) failure of ≥1 month of antiarrhythmic therapy or documented contraindication to prolonged medical management. The exclusion criteria were as follows: (1) AT not originating from the atrial appendage; (2) structural congenital heart disease requiring concurrent surgical repair (other than appendage-related pathology); and (3) incomplete clinical records.

### 2.2 P-wave morphology classification

The AT origin was preliminarily localized by surface 12-lead ECG P-wave morphology and polarity (2, 3). (1) RAA origin: negative “W”-shaped P-wave in lead V_1_; positive P-waves in leads II, III, aVF, I, and aVL; (2) LAA origin: positive “M”-shaped P-wave in lead V_1_; positive P-waves in leads II, III, and aVF; negative P-waves in leads I and aVL. When P-wave morphology was atypical, intracardiac activation mapping was used for confirmation.

### 2.3 Diagnostic criteria for tachycardia-induced cardiomyopathy

TIC was defined as follows: (1) uncontrolled AT; (2) left ventricular dysfunction [left ventricular ejection fraction (LVEF) < 50% and/or FS < 28%], with or without cardiac chamber enlargement (1); and (3) improvement in left ventricular systolic function (LVEF ≥ 55%, FS ≥ 28%) following effective AT control (5, 6).

### 2.4 Treatment allocation

Treatment was non-randomly assigned based on the anatomical origin of the tachycardia focus identified by Carto3 mapping and multidisciplinary team assessment, with informed parental consent. Allocation principles were as follows: (1) for AAT originating from the appendage *base*, where catheter contact stability is relatively favorable, catheter-based ablation (RFCA or PFA) was the primary approach; (2) for AAT, with earliest activation confirmed at the appendage *tip*—characterized by complex pectinate muscle architecture, marked anatomical variability, and limited transmural energy delivery—parents were informed of the risks and benefits of both RFCA/PFA and one-stop appendage resection and chose the treatment strategy; and (3) for appendage aneurysm-associated AAT, surgical resection was recommended given the high risk and low efficacy of catheter ablation. In this cohort, five patients underwent appendage resection (including 1 after RFCA failure), and one underwent PFA.

### 2.5 Electrophysiologic study and catheter ablation

#### 2.5.1 Preoperative preparation

All antiarrhythmic medications were discontinued for a minimum of five half-lives prior to the procedure.

#### 2.5.2 Electrophysiologic study

Standard electrophysiologic studies were performed using the Carto3 system. For left-sided substrates, transseptal access was achieved via the patent foramen ovale or under fluoroscopic guidance. A decapolar (DECA) catheter, a His bundle catheter, and a quadripolar catheter were positioned in the coronary sinus, high right atrium, and right ventricle, respectively, for simultaneous surface ECG and intracardiac electrogram recordings.

#### 2.5.3 Earliest activation site and focal origin criteria

Three-dimensional atrial reconstruction and activation mapping were performed using a Pentaray high-density mapping catheter (Biosense Webster), with ≥500 mapping points acquired per atrial chamber. The earliest activation site was defined as the location with the maximum bipolar electrogram-to-surface P-wave onset prematurity (20–40 ms), exhibiting a centrifugal radial activation pattern. To differentiate focal automaticity from micro-reentry, entrainment pacing was performed at and around the earliest activation site: (1) absence of stable fusion during entrainment; (2) post-pacing interval (PPI) minus tachycardia cycle length (TCL) > 30 ms; and (3) unipolar QS morphology at the target site. The combination of these features was considered suggestive of a focal mechanism (enhanced automaticity or triggered activity) (4, 7, 8).

#### 2.5.4 Radiofrequency catheter ablation

Irrigated radiofrequency energy was delivered at 17 mL/min, temperature ≤ 35 °C, and maximum power of 25–30 W. Acute success was defined as the simultaneous fulfillment of two criteria: (1) the immediate AT termination with restoration of sinus rhythm; and (2) the absence of spontaneous or inducible AT after 30 min of observation, including atrial programmed stimulation with and without isoproterenol infusion (0.1 µg·kg^−1^·min^−1^) (5, 9).

#### 2.5.5 Pulsed field ablation (PFA)

A second-generation PFA catheter (Pulsed FA FocalPoint; Jinjiang Electronics, China) was used at a preset voltage of 1,800 V (4/5 phase), with five superimposed pulses per application, and three total ablation sequences. PFA acute success was defined similar to that of RFCA (termination plus non-inducibility).

#### 2.5.6 Anticoagulation

Intravenous heparin (50 U/kg; maximum, 3,000 U) was administered for left-sided procedures, with a half-dose booster every 60 min. Postoperative aspirin (2–3 mg·kg^−1^·day^−1^; maximum 100 mg/day) was prescribed for 1 month (RAA) or 3 months (LAA).

### 2.6 Surgical technique

Under general anesthesia, the patient was placed in the lateral decubitus position. A 2–3 cm transaxillary incision was made through the fourth intercostal space. The pericardium was suspended to expose the target appendages. Clamping the appendage base immediately terminated AT, confirming the arrhythmia’s origin. The appendage was resected and submitted for histopathological examination, and the proximal stump was closed with a running 5–0 Prolene suture. A chest drain was placed, and the thoracotomy was closed in layers. Cardiopulmonary bypass (CPB) or cardiac arrest was not required. Procedures in which Carto3 mapping and appendage resection were performed under the same general anesthesia were designated “one-stop” procedures.

### 2.7 Complications, follow-up, and recurrence

Complications were defined as adverse events directly attributable to the intervention requiring additional intervention, prolonging hospitalization, or affecting long-term prognosis, including second- or third-degree atrioventricular block, cardiac perforation, pericardial effusion requiring drainage, thromboembolism, phrenic nerve injury, stroke, and cardiac arrest.

#### 2.7.1 Follow-up protocol

All patients underwent echocardiography and 12-lead ECG within 1–2 days postoperatively. Outpatient follow-up was scheduled at 1, 3, 6, and 12 months, and every 6–12 months thereafter, with a 12-lead ECG at each visit and 24 h Holter monitoring when recurrence was suspected. The minimum follow-up period for inclusion in the outcome analysis was 6 months (Case 3).

Recurrence was defined as sustained AT (≥30 s) with P-wave morphology identical to the pre-procedure baseline documented ≥5 days postoperatively by a 12-lead ECG or 24 h Holter, confirmed independently by ≥2 electrophysiologists (5, 9).

#### 2.7.2 Recurrence-free survival

All five patients with appendage resection maintained sinus rhythm throughout follow-up (recurrence-free survival equal to follow-up duration). The patient with PFA had a recurrence-free interval of 1 month. Given the small sample size and variable follow-up, formal survival analysis was not performed; the data are presented descriptively (Table 3).

### 2.8 Statistical analysis

Normally distributed continuous data are presented as mean ± SD; non-normally distributed data as median (IQR); and categorical data as frequency (percentage). Given the very small overall sample size (*n* = 6) and the TIC subgroup of only three patients, all statistical results were descriptive and hypothesis-generating rather than inferential, and no intergroup comparisons were performed. The Friedman test was used to describe temporal trends in cardiac function across five time points in patients with TIC; Friedman *P* values are reported for descriptive reference only and do not represent inferential conclusions. Analyses were performed using SPSS 27.0 (IBM Corporation, Armonk, NY, USA).

## 3 Results

### 3.1 Patient screening and allocation

Of the 27 children who underwent Carto3-guided electrophysiologic study for AT during the study period, 21 were excluded (non-appendage origin), and 6 (22.2%) were confirmed to have AAT and were enrolled (Figure 1). Five patients (Cases 1–5) underwent transaxillary appendage resection (Case 1 after initial RFCA failure) and one (Case 6) underwent PFA. The screening and allocation pathways are shown in Figure 1.

**Figure 1:**
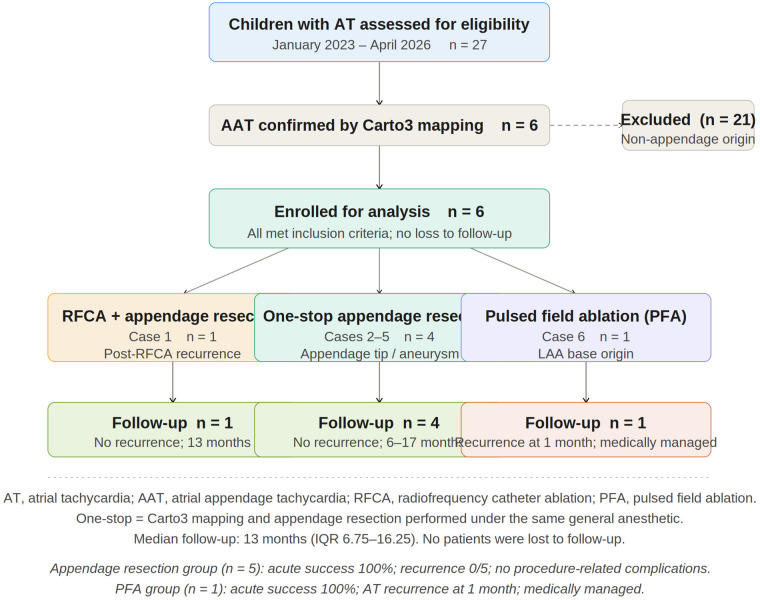
Patient screening and allocation flow diagram. Of the 27 children assessed, 6 (22.2%) had confirmed AAT and were enrolled; 5 underwent appendage resection, and 1 underwent PFA.

### 3.2 Baseline characteristics

Six children were enrolled: four males (66.7%) and two females (33.3%). The mean age was 10.28 ± 3.49 years (range: 3.7–14.0), the mean height was 149.3 ± 23.6 cm, and the mean weight was 39.6 ± 14.4 kg. All patients were symptomatic: palpitations in 5 (83.3%), chest tightness in 3 (50%), and fatigue with reduced exercise tolerance in 1 (16.7%). Five (83.3%) had incessant AT (mean heart rate 134 ± 26.4 bpm). Three patients were initially misdiagnosed [Case 1 as supraventricular tachycardia (SVT); Cases 2 and 4 as sinus tachycardia]. All patients had received ≥1 month of antiarrhythmic therapy without an adequate response. Three patients (50%) had TIC; the median preoperative LVEF was 49% (IQR 37.2%–53%), and the median BNP was 3,571.5 (IQR 451.5–5,257.5) pg/mL. Case 5 had concurrent left ventricular non-compaction (LVNC). The detailed baseline characteristics are presented in Table 1.

**Table 1:** Baseline clinical characteristics (*n* = 6).

No.	Sex	Age (years)	Height (cm)	Weight (kg)	Initial Dx	Symptoms	Pre-op drugs	AT pattern	HR (bpm)	BNP (pg/mL)	Pre-op EF (%)
1	F	9	137	29	SVT	Palp. 8 months	Met, Sot	Incessant	137	438	67
2	F	11	164	38.9	Sinus tachy	Palp. 2 months	Sotalol	Incessant	139	821	53
3	M	14	170	50.3	AT	Chest tight. 1 week	None	Paroxysmal	173	456	67
4	M	10	145	40	Sinus tachy	Chest tight. 10 days	Met, Prop	Incessant	147	623	49
5	M	3.7	110	19.5	AT	Fatigue 1 year	Sotalol	Incessant	98	18,567	37.2
6	M	14	170	60	AT	Chest tight. + palp. 4 days	None	Incessant	112	524	67

SVT, supraventricular tachycardia; Met, metoprolol; Sot, sotalol; Prop, propafenone; Palp., palpitations; tachy, tachycardia.

### 3.3 Electrophysiologic mapping

Carto3 activation mapping demonstrated centrifugal radial spread of the earliest activation in all six patients, with an activation time window substantially shorter than the tachycardia cycle length. Entrainment pacing in the five evaluable patients showed no stable fusion and PPI−TCL >30 ms, with unipolar QS morphology at the target—features suggestive of a focal origin. The earliest activation was at the appendage tip in five patients (83.3%) and at the base in one patient (16.7%); three patients had an LAA origin (Cases 1 and 2 at the LAA tip; Case 6 at the LAA base), and three patients had an RAA origin (Cases 3, 4, and 5 at the RAA tip). Representative surface ECG P-wave morphologies of RAA and LAA tachycardia are shown in Figure 2. In Case 5, intraoperative confirmation of an RAA aneurysm was made, and Carto3 showed diffuse earliest activation consistent with complex aneurysmal anatomy (Figure 3). Case 1 underwent RFCA with acute success but developed Holter-confirmed recurrence on postoperative day 5 and subsequently underwent appendage resection. Case 6 underwent PFA with acute success but recurred at 1 month and is currently being managed medically (last LVEF 69%).

**Figure 2:**
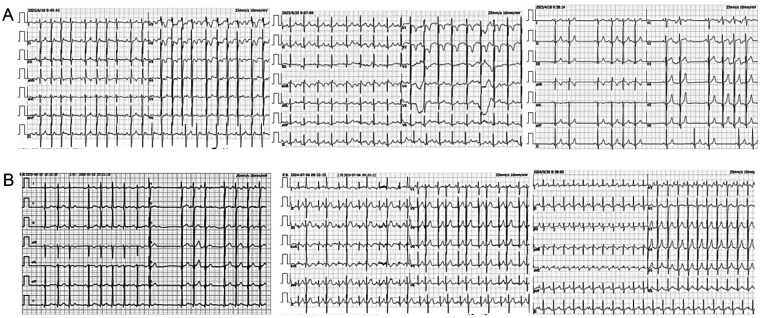
**(A)** Surface ECG of RAA tachycardia: negative “W”-shaped P-wave in V_1_; positive P-waves in II, III, aVF, I, and aVL. **(B)** Surface ECG of LAA tachycardia: positive “M”-shaped P-wave in V_1_; positive P-waves in II, III, aVF; and negative P-waves in I and aVL.

**Figure 3:**
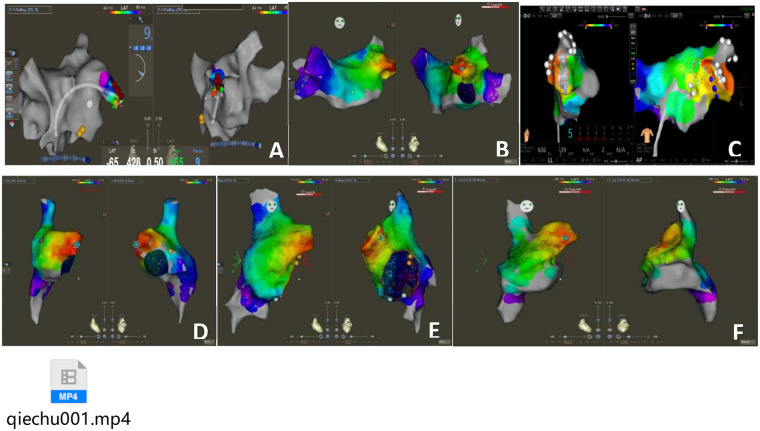
**(A,B)** Carto3 activation maps of LAA tip AT (RAO 30° and LAO 45°). **(C)** Earliest activation at the LAA base; pattern suggestive of focal origin. **(D–F)** Carto3 maps of the RAA tip AT. **(E inset):** RAA aneurysm with diffuse earliest activation.

### 3.4 Appendage resection group (*n* = 5)

Five patients (Cases 1–5) underwent minimally invasive transaxillary appendage resection. Case 1 was performed after RFCA failure, and Cases 2–5 underwent one-stop resection. In all five patients, appendage clamping immediately terminated AT with sinus rhythm restoration (acute success 5/5). The operative duration ranged from 50 to 76 min (median: 60 min). Ventilator support was required for 50 to 125 min, while the ICU stay ranged from 16 to 22 h. The intraoperative blood loss was between 10 and 30 mL. Cardiopulmonary bypass was not required, and no major procedure-related complications were observed. Detailed surgical data are provided in Table 2. Intraoperative findings and resected specimens are shown in Figure 4. Histopathological examination of the resected specimens is presented in Figure 5.

**Table 2:** Intraoperative data for appendage resection (*n* = 5).

Case	Appendage site	Op time (min)	Ventilator (min)	ICU (h)	Blood loss (mL)	CPB	Complications
1	LAA	50	95	22	10	No	None
2	LAA	60	125	16	10	No	None
3	RAA	75	85	20	20	No	None
4	RAA	76	50	18	30	No	None
5	RAA aneurysm	60	75	16	10	No	None

CPB, cardiopulmonary bypass; LAA, left atrial appendage; RAA, right atrial appendage.

**Figure 4:**
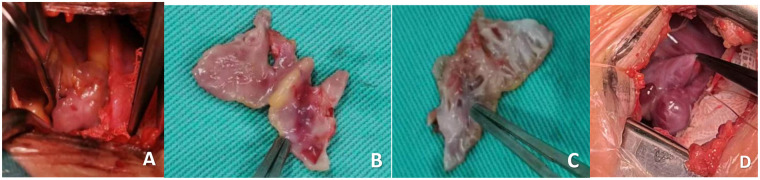
**(A)** Intraoperative view of the LAA apex (chicken wing morphology). **(B,C)** Resected LAA specimens with visible RF ablation lesions. **(D)** Aneurysmal RAA dilatation with markedly thinned walls.

**Figure 5:**
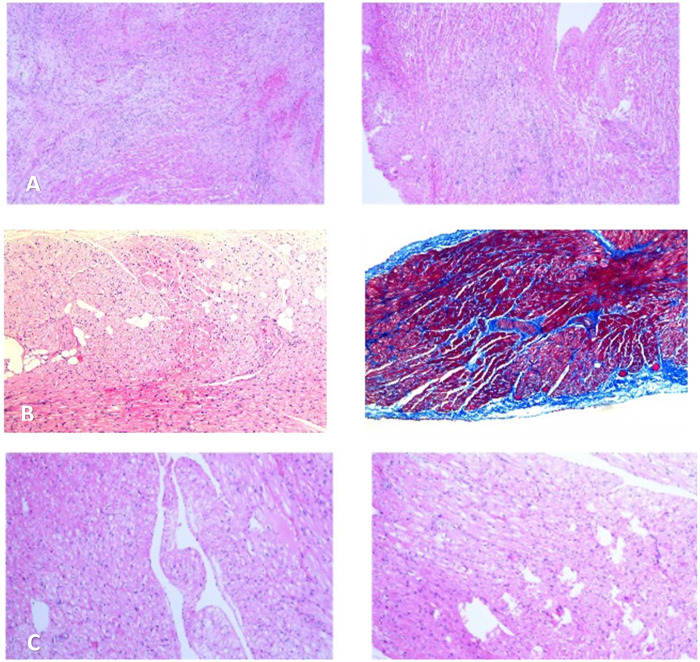
Histopathology. **(A)** LAA apex post-RFCA—focal myocardial necrosis and fibroblast proliferation. **(B)** LAA apex (no prior RFCA)—regularly arranged myocardial bundles and mild fibrosis. **(C)** RAA apex—cardiomyocyte hydropic degeneration and focal disarray.

### 3.5 Follow-up

The median follow-up was 13 months (IQR 6.75–16.25; minimum 6 months). All five patients who underwent appendage resection maintained sinus rhythm throughout the follow-up period (recurrence 0/5, 0%). Case 6 (PFA) experienced AT recurrence at 1 month and is currently being medically managed with stable cardiac function (LVEF 69%). The treatment and follow-up details are summarized in Table 3.

**Table 3:** Treatment and follow-up summary (*n* = 6).

No.	Procedure	Target	Acute success	Post-op EF (%)	F/U (months)	Last EF (%)	Recurrence/complications
1	RFCA + resection	LAA tip	Yes	62.3	13	70.7	No/none
2	One-stop resection	LAA tip	Yes	61.5	16	65.0	No/none
3	One-stop resection	RAA tip	Yes	69.0	6	69.5	No/none
4	One-stop resection	RAA tip	Yes	55.3	17	66.0	No/none
5	One-stop resection	RAA aneurysm	Yes	37.8	13	55.0	No/LVNC
6	PFA	LAA base	Yes	69.0	7	69.0	1 month/none

RFCA, radiofrequency catheter ablation; PFA, pulsed field ablation; LVNC, left ventricular non-compaction; F/U, follow-up. One-stop = Carto3 mapping and resection under the same anesthetic.

### 3.6 Cardiac function in patients with TIC (descriptive)

In the three patients with TIC (Cases 2, 4, and 5), left ventricular function showed a gradually improving trend following appendage resection (Table 4; Figure 6). These data are presented descriptively; given *n* = 3, the statistical power was insufficient for inferential conclusions. The LVEF increased from 46.3 ± 8.3% preoperatively to 62.0 ± 6.1% at the final follow-up. FS improved from 23.3 ± 4.8% to 33.3 ± 4.6%. Left ventricular end-diastolic diameter (LVEDd) decreased from 47.3 ± 3.5 mm to 42.0 ± 5.3 mm. In Case 5, with concurrent LVNC, there was a modest immediate improvement (LVEF 38.5%), but at the final follow-up, it reached 55.0%, suggesting that arrhythmia elimination may be the key determinant of functional recovery.

**Table 4:** Serial cardiac function in patients with TIC (*n* = 3, descriptive).

Parameter	Pre-op (a)	Immediately post-op (b)	Pre-discharge (c)	3-month F/U (d)	Final F/U (e)	Reference *P* value^a^
LVEF (%)	46.3 ± 8.3	51.5 ± 12.3	55.6 ± 8.4	59.6 ± 7.9	62.0 ± 6.1	0.022
FS (%)	23.3 ± 4.8	26.5 ± 7.5	28.9 ± 5.3	31.5 ± 5.2	33.3 ± 4.6	0.022
LVEDd (mm)	47.3 ± 3.5	44.8 ± 2.1	43.0 ± 3.5	41.4 ± 4.0	42.0 ± 5.3	0.140

a Reference *P* values from the Friedman test; given *n* = 3, the statistical power is extremely limited. These values are for the descriptive trend reference only and do not represent inferential conclusions. LVEF, left ventricular ejection fraction; FS, fractional shortening; LVEDd, left ventricular end-diastolic diameter.

**Figure 6:**
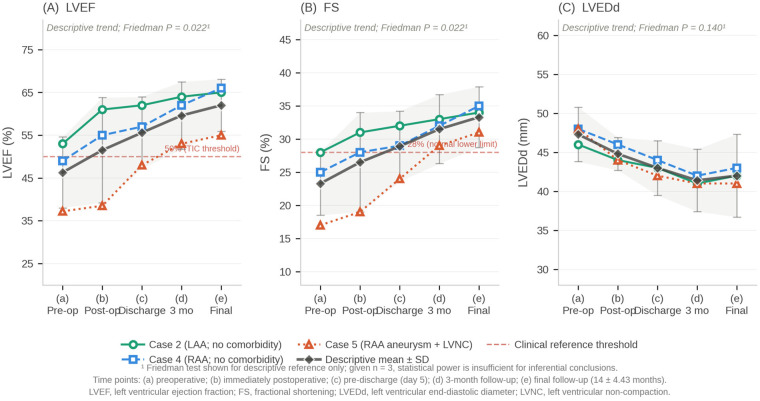
Descriptive serial cardiac function trends in 3 patients with TIC after appendage resection (individual trajectories and mean ± SD; *n* = 3, no inferential interpretation). **(A)** LVEF, **(B)** FS, and **(C)** LVEDd. Time points: **(a)** preoperative; **(b)** immediately postoperative; **(c)** pre-discharge (day 5); **(d)** 3-month follow-up; and **(e)** final follow-up (14 ± 4.43 months).

## 4 Discussion

This study reports the treatment experience of six pediatric patients with Carto3-confirmed AAT at a tertiary pediatric cardiac center. The principal findings were as follows: (1) AAT carries a high initial misdiagnosis rate and substantial TIC burden; (2) RFCA for appendage tip AAT has notable failure and recurrence rates, whereas transaxillary appendage resection showed initial feasibility in this small series; and (3) cardiac function in three patients with TIC showed a descriptive improving trend after arrhythmia elimination, although these observations require cautious interpretation.

### 4.1 Diagnostic challenges and TIC reversibility

AAT is clinically underrecognized, as evidenced by the fact that 50% of patients in this cohort were initially misdiagnosed. Clinicians should maintain a high index of suspicion for AAT with concurrent TIC in children presenting with incessant tachycardia, abnormal P-wave morphology, reduced LVEF, or increased LVEDd. The TIC prevalence of 50% is consistent with published reports (44.3%) (3). In the three patients with TIC, the LVEF improved from a mean of 46.3% to 62.0% at the final follow-up, and FS improved from 23.3% to 33.3%. These descriptive trends are consistent with prior reports of TIC reversibility (1, 3, 5); however, given *n* = 3 with substantial data dispersion (e.g., immediate post-op SD = 12.3%), individual variability cannot be excluded, and conclusions remain uncertain. In Case 5, with concurrent LVNC, there was a delayed recovery but ultimately achieved near-normal function, suggesting that arrhythmia elimination is a key determinant.

### 4.2 Limitations of RFCA for appendage tip AT

RFCA is the first-line therapy for pediatric AT (9). However, for AAT originating from the appendage tip, acute failure rates are 27.1%, post-ablation recurrence rates are 25.5%, and 45.7% of cases ultimately require surgical resection (3). Case 1 experienced recurrence on day 5 despite a technically successful RFCA. Anatomical reasons include (1) pectinate muscles impeding stable catheter contact; (2) thin, variable appendage walls increasing perforation risk (10); (3) RF energy attenuation by pectinate muscles; and (4) epicardial substrate location (4, 7). These considerations support the prioritization of surgical resection for appendage tip AAT in selected pediatric patients.

### 4.3 Comparison with standard treatments

Surgical appendage resection for AT was first described by de Bakker et al. in 1994 (11). Multiple studies have confirmed its potential for durable control of arrhythmia (12). The transaxillary approach offers (1) no sternotomy with superior cosmesis; (2) no cardiopulmonary bypass; (3) intraoperative clamping confirming arrhythmia origin; and (4) one-stop integration with Carto3 mapping. In this series, all five patients maintained sinus rhythm with no recurrence observed at a median 13-month follow-up, which preliminarily supports the feasibility of the approach (1, 3).

#### 4.3.1 Minimally invasive transaxillary appendage resection versus repeat RFCA and medical therapy

Repeat RFCA faces the same anatomical obstacles, resulting in cumulative recurrence rates of 25.5% (3). Long-term antiarrhythmic drug therapy (sotalol, propafenone) has limited efficacy for incessant AAT in children and carries the risks of proarrhythmia and QT prolongation. All patients in this cohort had failed ≥1 month of antiarrhythmic therapy, underscoring the need for a definitive treatment.

#### 4.3.2 Minimally invasive transaxillary appendage resection versus VATS

VATS requires double-lumen intubation and single-lung ventilation, along with specialized instruments that offer limited adaptability for young, low-weight children. The transaxillary approach uses standard pediatric cardiac surgical instruments and avoids the need for a second anesthesia when combined with Carto3 mapping.

### 4.4 Generalizability

The adoption of this technique requires (1) a pediatric electrophysiology team proficient in Carto3 mapping; (2) a pediatric cardiac surgical team experienced in minimally invasive approaches; and (3) a standardized perioperative management system. The relatively low resource threshold (no CPB; blood loss, 10–30 mL; ICU stay, 16–22 h) may facilitate adoption, but single-center experience limits external generalizability. A prospective multicenter evaluation is warranted before widespread implementation.

Long-term considerations include impaired ANP secretion after LAA resection, which may affect sodium–water homeostasis (7), and a theoretical stroke risk reduction offset by a potential increase in left atrial stiffness (13). The long-term effects of growing children remain incompletely characterized.

### 4.5 RAA aneurysm

RAA aneurysm (RAAA) is exceedingly rare (<44 reported cases as of 2025) (8, 14). Associated atrial arrhythmias occur in 30%–50% of cases (14). Owing to the proximity to the sinoatrial node and phrenic nerve, RFCA carries a significant risk. In Case 5, Carto3 showed diffuse earliest activation, consistent with the complex aneurysmal anatomy. Published histopathology of RAAA demonstrates wall thinning, cystic dilatation, fibrosis, and cardiomyocyte atrophy (3, 14, 15). The surgical resection resulted in immediate AT termination with no recurrence observed at 13 months, which supports resection as the preferred treatment for RAAA-associated AAT.

### 4.6 PFA: preliminary experience

PFA selectively induces irreversible electroporation while minimizing collateral injury (4). Urbanek et al. (16) first reported PFA for adult RAA tachycardia, whereas Nie et al. (7) described PFA for RAA tachycardia in a 5-year-old child. A systematic review of 312 patients with SVT reported 97.6% acute PFA success and 21.4% AT recurrence (4). Case 6 represents the first PFA application for pediatric LAA base tachycardia at our center. Despite the acute success, recurrence at 1 month limits the conclusions from this single case, and larger prospective studies are needed.

## 5 Conclusions

In this small preliminary series, one-stop minimally invasive transaxillary appendage resection demonstrated initial feasibility for pediatric AAT with a Carto3-confirmed appendage tip origin or an associated aneurysm refractory to medical therapy. Five patients maintained sinus rhythm at a median 13-month follow-up without major complications. Given the very small sample size (*n* = 5), the single-center non-randomized design, and limited follow-up, these findings provide preliminary feasibility evidence and cannot yet establish the procedure as “safe and effective.” Cardiac function in three patients with TIC showed a descriptive improving trend, suggesting possible TIC reversibility, but *n* = 3 precludes statistical inference. Validation in larger, multicenter, prospective studies is essential. The PFA in pediatric AAT warrants further investigation.

## 6 Study limitations

This study has several important limitations. (1) Very small sample size and single-center retrospective design: only six patients with AAT (surgical group *n* = 5; PFA *n* = 1) were enrolled, substantially limiting the statistical power and external generalizability. (2) Mixed, non-randomized treatment allocation: three treatment pathways were included, and intergroup comparison was not performed. (3) Short and variable follow-up: A median follow-up of 13 months (range: 6–17 months) is insufficient to assess the long-term complications of appendage resection or longitudinal cardiac function in growing children. (4) TIC subgroup *n* = 3: cardiac function data were descriptive only and did not support any inferential statistical conclusions. (5) Single PFA case: the efficacy of this modality cannot be assessed. These limitations indicate that all findings should be viewed as preliminary feasibility evidence that requires prospective multicenter validation.

## Data Availability

The original contributions presented in this study are included in the article/Supplementary Material. Further inquiries can be directed to the corresponding author.
